# Thyroid status and mortality in nonagenarians from long-lived families and the general population

**DOI:** 10.18632/aging.101310

**Published:** 2017-10-25

**Authors:** Nicolien A. van Vliet, Evie van der Spoel, Marian Beekman, P. Eline Slagboom, Gerard Jan Blauw, Jacobijn Gussekloo, Rudi G.J. Westendorp, Diana van Heemst

**Affiliations:** ^1^ Department of Internal Medicine, section of Gerontology and Geriatrics, Leiden University Medical Center, Leiden, 2300 RC, the Netherlands; ^2^ Department of Medical Statistics and Bioinformatics, section of Molecular Epidemiology, Leiden University Medical Center, Leiden, PO Box 9600, 2300 RC, the Netherlands; ^3^ Department of Public Health and Primary Care, Leiden University Medical Center, Leiden, 2300 RC, the Netherlands; ^4^ Department of Public Health and Center for Healthy Aging, University of Copenhagen, Copenhagen, Hovedstaden, 1014, Denmark

**Keywords:** thyroid, mortality, nonagenarians, longevity, familial

## Abstract

The relationship between thyroid status and longevity has been investigated extensively. However, data on thyroid status and survival in old age is scarce. In this study we investigated associations of different parameters of thyroid status with mortality in nonagenarians, and whether these associations were different in nonagenarians from long-lived families than in nonagenarians from the general population. In total, 805 nonagenarians from the Leiden Longevity Study and 259 nonagenarians from the Leiden 85-plus Study were followed up to collect mortality data. At baseline, levels of thyrotropin (TSH), free thyroxine (fT4) and free triiodothyronine (fT3) were measured. In nonagenarians from long-lived families and from the general population, associations between thyroid parameters and mortality were similar. We found no interaction between study population and parameters of thyroid status on mortality (P-values>0.70). The results from both studies were combined to derive generalizable associations. Hazard ratios (HRs) for the highest compared to lowest tertiles were determined, resulting in TSH HR 0.91 (P=0.25), fT4 HR 1.22 (P=0.02), fT3 HR 0.74 (P=1.31e-4), and fT3/fT4 HR 0.66 (P=5.64e-7). In conclusion, higher fT3/fT4 ratios, higher levels of fT3, and lower levels of fT4 were associated with lower mortality rate in nonagenarians and independent of familial longevity status.

## INTRODUCTION

Thyroid hormone is crucial for growth and development in every stage of life. Without proper tuning of thyroid hormone activity, adverse effects on cognitive function, bone mineralisation, and energy metabolism are common. In case of thyroid hormone deficiency (hypothyroidism), weight gain, mood impairment, and dyslipidaemia are prevalent problems [1]. Thyroid hormone excess (hyperthyroidism) may lead to atrial fibrillation, muscle wasting, weight loss and osteoporosis [2, 3]. Even within the normal range (euthyroidism), variations in thyroid status have been associated with cardiovascular risk and body composition [4].

Circulating thyroid hormone levels are regulated by the hypothalamus-pituitary-thyroid (HPT) axis [5]. First the hypothalamus secretes thyrotropin releasing hormone (TRH), inducing release of thyrotropin (TSH) by the pituitary. TSH then stimulates the thyroid gland to synthesise and secrete thyroid hormones, consisting of the prohormone thyroxine (T4) and the active hormone triiodothyronine (T3). Most of T4 and T3 is bound to binding proteins, resulting in only a small fraction of unbound, free T4 (fT4) and unbound, free T3 (fT3) in the circulation. Subsequently, through negative feed-back, the hypothalamus and pituitary gland adjust secretion of TRH and TSH to circulating levels of fT4. In peripheral tissues, fT4 is converted by tissue specific deiodinases to customize local availability of active T3. Previously, indications were found of a relationship between lower thyroid status and longevity. In the Leiden 85-plus Study, a prospective population-based study in the city of Leiden in the Netherlands [6], higher levels of TSH were associated with lower mortality in participants between ages 85 and 90 [7]. Furthermore, in the Leiden Longevity Study, a family study of siblings with exceptional longevity [8, 9], slightly higher levels of circulating TSH and lower levels of fT4 and fT3 were associated with increased familial longevity [10]. The offspring of these old age siblings had higher TSH levels than their partners, while they had similar levels of fT4 and fT3 [11]. Taken together, these studies suggest a different HPT-axis set point as a familial trait of long-lived families. However, data on associations between thyroid status and survival at exceptionally old age are scarce and no data are available on whether these associations are similar in nonagenarians from long-lived families as compared to nonagenarians from the general population.

To address these questions, we assessed and compared the associations between parameters of thyroid status and mortality in two nonagenarian study populations; the Leiden Longevity Study and the Leiden 85-plus Study.

## RESULTS

### Characteristics of study populations

Characteristics of the nonagenarian participants of the Leiden Longevity Study and the Leiden 85-plus Study are presented separately for the 384 men and 680 women in Table 1. For the Leiden Longevity Study, male participants were younger (91.4 years) than the female participants (93.6 years, P<0.001). Circulating levels of fT3 were significantly higher in men (4.1 pmol/L) than in women (4.0 pmol/L, P=0.001). The women had lower fT3/fT4 ratios (0.25) compared to the men (0.26, P<0.001). The other parameters were not significantly different. In the Leiden 85-plus Study, measurements performed at age 90 years were taken as baseline for both men and women. Levels of high-sensitivity C-reactive protein (hsCRP) were significantly different between men and women (men 3.7 mg/L, women 2.8 mg/L, P=0.02). All other measured parameters were similar for men and women.

**Table 1:** Characteristics of the participants of the Leiden Longevity Study and the Leiden 85-plus Study

	Men	Women	P-value
**Leiden Longevity Study**			
Number of participants	312	493	
Age (years)	91.4 (90.1-93.7)	93.6 (92.2-95.3)	**<0.01**
Deceased (n (%))	287 (92.0)	462 (93.7)	0.88
TSH (mU/L)	1.6 (1.0-2.4)	1.5 (1.0-2.4)	0.16
fT4 (pmol/L)	15.8 (2.0)	16.1 (2.4)	0.08
fT3 (pmol/L)	4.1 (0.6)	4.0 (0.5)	**<0.01**
fT4xTSH (pmolxmU)	25.3 (16.3-36.2)	24.1 (15.6-37.5)	0.26
fT4/TSH (pmol/mU)	9.9 (6.2-14.9)	10.8 (6.3-17.2)	0.10
fT3/fT4	0.26 (0.05)	0.25 (0.04)	**<0.01**
hsCRP (mg/L)^a^	3.0 (1.4-6.6)	2.6 (1.2-5.2)	0.13
**Leiden 85-plus Study**			
Number of participants	72	187	
Age (years)	90	90	
Deceased (n (%))	71 (98.6)	183 (97.9)	0.16
TSH (mU/L)	1.9 (1.1-3.0)	1.7 (1.0-2.9)	0.46
fT4 (pmol/L)	16.2 (2.5)	16.3 (2.2)	0.80
fT3 (pmol/L)	4.0 (0.5)	4.1 (0.6)	0.27
fT4xTSH (pmolxmU)	30.5 (19.5-47.0)	26.9 (18.1-44.2)	0.41
fT4/TSH (pmol/mU)	8.4 (5.0-15.9)	9.2 (5.2-16.3)	0.49
fT3/fT4	0.25 (0.05)	0.26 (0.04)	0.94
hsCRP (mg/L)	3.7 (2.0-8.3)	2.8 (1.2-5.8)	**0.02**

Data are presented as median (interquartile range) or mean (standard deviation) where appropriate, unless indicated otherwise.

P-values shown for Mann-Whitney U test, T-Test, or Chi-Square test where appropriate.

^a^ data available for 312 men and 492 women.

### Thyroid status and mortality in long-lived families

In Table 2 the associations between thyroid status parameters and mortality rate in the participants of the Leiden Longevity Study are presented. For levels of TSH, no association with mortality rate was found. For levels of fT4, a more than 20 percent increased mortality rate was observed in the highest tertile compared to the lowest, however statistical significance was lost after correction for hsCRP (model 1 hazard ratio (HR) 1.22 (95% CI 1.01-1.48) P=0.04, model 2 HR 1.21 (95% CI 0.99-1.45) P=0.06). Higher levels of fT3 were associated with lower mortality in both models (model 1 HR 0.70 (95% CI 0.58-0.85) P=2.33×10^−4^, model 2 HR 0.73 (95% CI 0.60-0.88) P=1.33×10^−3^). The fT4xTSH product and the fT4/TSH ratio were not associated with mortality in either model. The highest tertile of the fT3/fT4 ratio had more than 30 percent reduction in mortality rate compared to the lowest tertile (model 1 HR 0.65 (95% CI 0.54-0.79) P=1.65×10^−5^, model 2 HR 0.68 (95% CI 0.56-0.83) P=1.13×10^−4^). The results were similar for men and women in the stratified analyses (data not shown).

**Table 2:** Parameters of thyroid status and mortality rate in the Leiden Longevity Study

Leiden Longevity Study participants						
	Median (range)		Hazard ratio (95% CI)			
	Men(n=312)	Women(n=493)	Model 1(n=805)	P-value	Model 2 ^a^ (n=804)	P-value
**TSH**						
Lowest tertile	0.9 (0.1-1.2)	0.7 (0.1-1.1)	1.00 (reference)		1.00 (reference)	
Middle tertile	1.6 (1.2-2.1)	1.5 (1.1-2.0)	0.92 (0.78-1.08)	0.30	0.91 (0.77-1.07)	0.24
Highest tertile	3.0 (2.1-11.7)	3.0 (2.0-17.3)	0.95 (0.79-1.14)	0.49	0.93 (0.77-1.12)	0.44
**fT4**						
Lowest tertile	13.8 (10.2-14.7)	13.7 (10.1-15.0)	1.00 (reference)		1.00 (reference)	
Middle tertile	15.6 (14.8-16.6)	16.0 (15.1-16.9)	0.91 (0.75-1.09)	0.30	0.92 (0.76-1.10)	0.36
Highest tertile	17.7 (16.7-22.1)	18.3 (17.0-23.5)	1.22 (1.01-1.48)	**0.04**	1.21 (0.99-1.45)	0.06
**fT3**						
Lowest tertile	3.6 (2.6-3.8)	3.5 (2.6-3.7)	1.00 (reference)		1.00 (reference)	
Middle tertile	4.1 (3.9-4.3)	3.9 (3.8-4.1)	0.77 (0.64-0.93)	**0.01**	0.78 (0.65-0.94)	**0.01**
Highest tertile	4.6 (4.4-5.8)	4.5 (4.2-5.8)	0.70 (0.58-0.85)	**<0.01**	0.73 (0.60-0.88)	**<0.01**
**fT4xTSH**						
Lowest tertile	14.0 (2.0-19.5)	12.3 (1.6-18.2)	1.00 (reference)		1.00 (reference)	
Middle tertile	25.3 (19.5-32.1)	24.1 (18.2-30.9)	1.00 (0.84-1.18)	0.96	0.99 (0.84-1.18)	0.93
Highest tertile	45.6 (32.2-212.9	46.9 (31.1-257.5)	1.01 (0.83-1.22)	0.92	1.01 (0.83-1.22)	0.94
**fT4/TSH**						
Lowest tertile	5.2 (1.2-7.5)	4.8 (0.6-7.8)	1.00 (reference)		1.00 (reference)	
Middle tertile	9.9 (7.5-13.0)	10.8 (7.8-14.3)	0.92 (0.77-1.10)	0.35	0.92 (0.77-1.10)	0.34
Highest tertile	18.6 (13.1-136.7)	22.5 (14.3-235.0)	1.07 (0.90-1.29)	0.44	1.08 (0.91-1.29)	0.38
**fT3/fT4**						
Lowest tertile	0.22 (0.14-0.24)	0.21 (0.12-0.23)	1.00 (reference)		1.00 (reference)	
Middle tertile	0.26 (0.24-0.28)	0.25 (0.23-0.27)	0.73 (0.61-0.88)	**<0.01**	0.75 (0.63-0.90)	**<0.01**
Highest tertile	0.31 (0.28-0.41)	0.29 (0.27-0.37)	0.65 (0.54-0.79)	**<0.01**	0.68 (0.56-0.83)	**<0.01**

Participants were divided over sex-specific tertiles, which were combined for analyses.

Model 1: adjusted for family number and age

Model 2: adjusted for family number, age, and hsCRP

^a^ data available for 312 men and 492 women.

### Thyroid status and mortality in the general population

The association between thyroid status parameters and mortality rate in participants of the Leiden 85-plus Study are presented in Table 3. Similar to the results in the participants of the Leiden Longevity Study, levels of TSH were not associated with mortality. Higher levels of fT4 were associated with an increased mortality rate in both models, though statistical significance was not reached (model 1 HR 1.36 (95% CI 1.00-1.84) P=0.05, model 2 HR 1.27 (95% CI 0.93-1.73) P=0.14). The highest tertile of fT3 had a significantly lower mortality rate than the lowest tertile (model 1 HR 0.66 (95% CI 0.49-0.90) P=0.01, model 2 HR 0.72 (95% CI 0.53-0.98) P=0.04) The fT4xTSH product and the fT4/TSH ratio were not associated with mortality rate. The fT3/fT4 ratio was associated with a more than 40 percent decrease in mortality rate in both models in the highest compared to the lowest tertile (model 1 HR 0.55 (95% CI 0.40-0.75) P=1.44×10^−4^, model 2 HR 0.59 (95% CI 0.43-0.81) P=9.66×10^−4^). The results were similar for men and women in the stratified analyses (data not shown).

**Table 3:** Parameters of thyroid status and mortality rate in the Leiden 85-plus Study

Leiden 85-plus Study participants						
	Median (range)		Hazard ratio (95% CI)			
	Men(n=72)	Women(n=187)	Model 1(n=259)	P-value	Model 2 (n=259)	P-value
**TSH**						
Lowest tertile	0.9 (0.1-1.3)	0.8 (0.1-1.2)	1.00 (reference)		1.00 (reference)	
Middle tertile	1.9 (1.4-2.7)	1.7 (1.3-2.4)	0.87 (0.64-1.18)	0.38	0.91 (0.67-1.23)	0.53
Highest tertile	3.9 (2.7-7.5)	3.5 (2.4-14.6)	0.86 (0.64-1.17)	0.35	0.87 (0.64-1.17)	0.35
**fT4**						
Lowest tertile	13.8 (11.5-14.6)	14.3 (11.2-15.3)	1.00 (reference)		1.00 (reference)	
Middle tertile	16.2 (14.7-17.4)	16.2 (15.4-17.1)	1.37 (1.01-1.86)	0.05	1.35 (0.99-1.83)	0.06
Highest tertile	18.9 (17.5-22.2)	18.3 (17.2-23.2)	1.36 (1.00-1.84)	0.05	1.27 (0.93-1.73)	0.14
**fT3**						
Lowest tertile	3.6 (2.7-3.8)	3.5 (2.8-3.8)	1.00 (reference)		1.00 (reference)	
Middle tertile	4.0 (3.9-4.1)	4.0 (3.9-4.3)	0.77 (0.57-1.06)	0.11	0.82 (0.60-1.12)	0.21
Highest tertile	4.4 (4.2-5.7)	4.7 (4.4-5.9)	0.66 (0.49-0.90)	**0.01**	0.72 (0.53-0.98)	**0.04**
**fT4xTSH**						
Lowest tertile	15.0 (1.8-23.1)	13.7 (2.0-20.1)	1.00 (reference)		1.00 (reference)	
Middle tertile	30.5 (23.2-42.7)	26.9 (20.5-39.0)	1.21 (0.89-1.64)	0.23	1.24 (0.91-1.69)	0.17
Highest tertile	57.4 (43.8-108.2)	57.5 (39.0-210.5)	0.98 (0.72-1.33)	0.89	0.98 (0.72-1.33)	0.88
**fT4/TSH**						
Lowest tertile	3.5 (1.8-5.9)	4.6 (0.8-6.4)	1.00 (reference)		1.00 (reference)	
Middle tertile	8.4 (6.1-12.0)	9.2 (6.4-13.7)	0.97 (0.72-1.32)	0.86	1.01 (0.74-1.36)	0.96
Highest tertile	17.3 (12.6-181.0)	21.1 (13.7-165.8)	1.12 (0.83-1.52)	0.46	1.12 (0.83-1.52)	0.46
**fT3/fT4**						
Lowest tertile	0.21 (0.13-0.23)	0.22 (0.14-0.24)	1.00 (reference)		1.00 (reference)	
Middle tertile	0.25 (0.23-0.27)	0.26 (0.24-0.27)	0.73 (0.54-0.98)	**0.04**	0.76 (0.56-1.03)	0.07
Highest tertile	0.30 (0.28-0.37)	0.29 (0.27-0.36)	0.55 (0.40-0.75)	**<0.01**	0.59 (0.43-0.81)	**<0.01**

Participants were divided over sex-specific tertiles, which were combined for analyses.

Model 1: crude analysis

Model 2: adjusted for hsCRP

### Thyroid status and mortality independent of study population

The results of the stratified analyses on thyroid status and mortality were similar for both nonagenarians of long-lived families and of the general population. When interaction was tested between study population and parameters of thyroid status on mortality, no significant interaction was found (all P-values>0.7, Supplementary Table 1).

### Generalized results for nonagenarians

**Figure 1:**
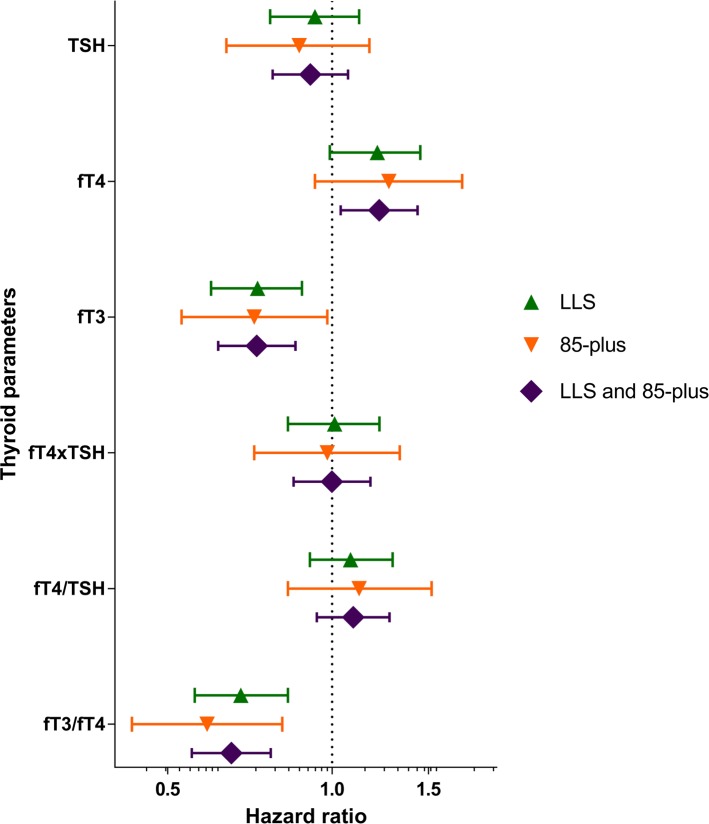
Mortality rate in the Leiden Longevity Study and the Leiden 85-plus Study Hazard ratios and 95% confidence intervals for mortality of the highest sex-specific tertiles compared to the lowest sex-specific tertiles are shown for the Leiden Longevity Study participants, the Leiden 85-plus Study participants and the pooled Hazard ratios. LLS = participants of the Leiden Longevity Study, 85-plus = participants of the Leiden 85-plus Study, LLS and 85-plus = combined estimates of the Leiden Longevity Study and the Leiden 85-plus Study.

Since the associations between thyroid status and mortality were similar in both nonagenarian popu-lations, the results could be combined to provide a generalizable association for nonagenarians. For this purpose, the results for the Leiden Longevity Study and the Leiden 85-plus Study populations were combined, through a fixed-effects inverse-variance weighted analysis on the associations of thyroid parameters and mortality rate with adjustments as described in model 2 (Supplementary Table 2). The HRs of the highest compared to the lowest tertile in both studies and the weighted averages of these HRs are displayed in Figure 1. Higher levels of TSH were not associated with mortality rate in either study or in the pooled analysis (HR 0.91, 95% CI 0.78-1.07, P=0.25). Higher levels of fT4 were associated with higher mortality rate in both studies, leading to an increase of 22 percent in mortality rate in the highest compared to the lowest fT4 tertile in the pooled analysis (HR 1.22, 1.04-1.43, P=0.02).

Inversely, higher levels of fT3 were associated with lower mortality rates in both studies, resulting in a decrease in mortality rate of more than 25 percent in the highest compared to the lowest tertile (HR 0.73, 95% CI 0.62-0.86, P=1.31×10^−4^). The fT4xTSH product and the fT4/TSH ratio were not associated with mortality rate. Lastly, the fT3/fT4 ratio was associated with a lower mortality rate in the highest tertiles compared to the lowest in both studies, resulting in a 35 percent lower mortality rate in the pooled analysis (HR 0.65, 95% CI 0.55-0.77, P=5.64×10^−7^).

### Various sensitivity analyses

Sensitivity analyses performed on participants with normal levels of hsCRP (hsCRP<10 mg/L), participants with mortality within the first two years or past the first year of follow up did not materially change the results (data not shown). The results were similar in participants with TSH levels within the normal range as in the main analyses (data not shown).

## DISCUSSION

This study assessed the association between parameters of thyroid status and mortality rate in nonagenarians from long-lived families and the general population. In both populations, a higher fT3/fT4 ratio was associated with decreased mortality rate. Although the observed associations for the levels of fT3 and fT4 within the reference range were not identical for the two populations, these showed a similar pattern. When the results of both populations were combined, higher levels of fT3 within the reference were associated with a lower mortality rate, whereas higher levels of fT4 within the reference range were detrimental in old age. Risk of mortality was neither associated with levels of TSH nor with the fT4xTSH product or the fT4/TSH ratio. All observed relationships between thyroid status and risk of mortality were consistent in male and female participants and in participants with TSH levels within the normal range.

The fT3/fT4 ratio can be viewed as an estimate of conversion of fT4 into fT3 [12]. Conversion of fT4 takes place by deiodinases in peripheral tissues such as the liver, the kidneys and skeletal muscle as well as inside the thyroid gland [13]. Our result of a lower mortality rate in the participants with the highest fT3/fT4 ratios in nonagenarians is consistent with the previous finding of Gussekloo et al. [7] in participants of the Leiden 85-plus Study aged 85 to 89 years old. One of the possible explanations for this association is that individuals with a lower fT3/fT4 ratio are ill. In times of critical illness and inflammation, conversion of T4 to T3 is inhibited by downregulation of type 1 deiodinase (D1) known as euthyroid sick syndrome [14]. However, when we adjusted for levels of C-reactive protein as a marker of inflammation, the observed association was not attenuated.

The increased risk of mortality with higher levels of fT4 could be a reflection of lower turnover. This could be due to decreased conversion of fT4 by deiodinases, congruent to the observation with the fT3/fT4 ratio. Alternatively, higher circulating levels of fT4 might reflect a slower rate of detoxification of thyroid hormones (and possibly other compounds) by the liver and kidney which would be unfavourable for survival. These associations are in line with previous observations [7, 15-18].

The association between higher circulating levels of fT3 and lower mortality is in line with previous research. A higher BMI was found to be causally related to higher circulating levels of fT3 [19], and higher BMI has also been positively associated with old age survival [20]. The lower risk of mortality with higher levels of fT3 we found was reported by others only in crude analyses when illness was not taken into account [18, 21, 22]. Yet, adjustment for levels of hsCRP did not materially influence the results.

Circulating levels of TSH appear to play no major role in risk of mortality in our nonagenarian populations, which matches the inconclusive results in literature [7, 15, 17, 22-27]. In previously described analyses, decreased mortality with increasing levels of TSH was observed in the prolonged follow-up of the 85-plus Study [5]. However, in participants with TSH levels within the normal range the decrease in mortality was not significant, which is in line with our study in which the participants with extreme values of TSH were excluded.

No relationship was found between mortality risk and the fT4xTSH product, which is a reflection of resistance to thyroid hormones due to thyroid hormone receptor defects [11, 28]. In mice, the influence on mortality depends on the affected receptor type. Defects in the β-receptor cause only marginal increase in mortality whereas α-receptor defects are associated with severely shortened lifespan [29]. Data on the effects of fT4xTSH in humans are scarce and no data is available for milder variations within populations.

The fT4/TSH ratio was also not associated with mortality rate in this study. This ratio combines the levels of fT4 and TSH, resulting in a composite measure which could reflect the responsiveness of the thyroid gland to TSH, a trait previously associated with familial longevity [10, 11, 30-32]. Possibly the influence of this trait is only found earlier in life, because selection for individuals with this trait has taken place by age 90 years.

The consistency of our results in both nonagenarian populations advocates a universal mechanism of survival in old age via relatively lower levels of fT4 and higher fT3. Even though nonagenarians from long-lived families exhibit demonstrably lower mortality rate than nonagenarians from the general population [9], a higher fT3/fT4 ratio appears to be a common trait associated with longevity.

One of the strengths of this study is that we used data from a relatively large population of a rarely investigated age category of nonagenarians. The study participants originated from two different study populations with different study designs and inclusion criteria. This has led to a high heterogeneity in study population, which allows extrapolation of these results to nonagenarians in the general population. Another quality of this study is the use of multiple parameters of thyroid status, which could offer more insight into the interplay of these parameters. These continuous values of proportions are preferable over clinical cut off points for clinical and subclinical thyroid dysfunction for two reasons. Firstly, these continuous variables allow for a more mechanistic approach. Secondly, the diagnostic criteria for thyroid dysfunction and normal ranges of TSH and fT4 differ over time, per country and per laboratory [33, 34]. Limitations of this study are the lack of information on thyroid disease and use of medication affecting the thyroid, as well as general information on chronic and acute illness.

In conclusion, we have found lower mortality rate associated with a higher fT3/fT4 ratio, lower fT4 levels, and higher fT3 levels in nonagenarians from long-lived families and from the general population. Based on the results of the present study, associations of thyroid status with old age mortality were not dependent on familial longevity status. To identify the causal mechanisms underlying the observed associations between thyroid status and mortality, future research should focus on biological mechanisms via which thyroid parameters might contribute to longevity.

## METHODS

### Leiden Longevity Study

As described previously in more detail, the aim of the Leiden Longevity Study is to identify genetic and phenotypical factors contributing to familial longevity [8, 9]. Families were included if at least two proband siblings had lived to exceptionally old age, being 89 years or older for men and 91 years or older for women. No exclusion criteria based on health or demographics were applied. The Medical Ethical Committee of the Leiden University Medical Centre approved the study and written informed consent was obtained from all study participants. Between July 2002 and May 2006, 421 Dutch Caucasian families were recruited, comprising 944 nonagenarian participants.

Of these 944 nonagenarians data on TSH, fT4 and fT3 were available for 859 participants. Three participants were excluded from the analyses because serum TSH levels were outside the detection range (two participants with TSH <0.005 mU/L and one participant with TSH >100mU/L). Four participants were excluded because their fT3 level was below the reference range (<2.5 pmol/L), as a possible sign of euthyroid sick syndrome. Lastly, 16 participants were excluded because their fT4 levels were outside the reference range (six participants with <10.0 pmol/L and ten participants with >24.0 pmol/L), because of presumed thyroid disease or medication use affecting the thyroid function. Subsequently, participants with (log transformed) TSH or fT4 or one of the composite measurements ((log transformed) fT4xTSH product, (log transformed) fT4/TSH ratio, or fT3/fT4 ratio) that deviated more than three standard deviations from the population mean were excluded. Of the included 805 participants, 749 had passed away on the 1^st^ of February 2014. The median follow up time was 3.49 years, with interquartile range of 1.53 to 4.97 years.

### Leiden 85-plus study

The Leiden 85-plus Study is a prospective, population-based study aimed at investigating determinants of successful aging [6]. Inclusion criteria were being 85 years old and living in Leiden, the Netherlands, between September 1997 and September 1999. No exclusion criteria based on health were applied. Out of 705 eligible individuals 599 participants were enrolled, since 92 people had refused and 14 were deceased before the assessment visit. Over a follow-up period of five years, participants were assessed annually until death or refusal. The study was approved by the Medical Ethical Committee of the Leiden University Medical Centre and all participants provided oral informed consent.

To investigate a similar age group as the Leiden Longevity Study, the current study used data of the participants of the Leiden 85-plus Study at the age of ninety years. Out of the 599 participants enrolled at 85 years, 52 had refused to continue participation and 270 were deceased before the assessment visit following their 90^th^ birthday. From the total of 277 nonagenarian participants who were eligible, three participants did not provide a blood sample and three had missing data on TSH level. One participant was excluded because the serum fT3 level was below the reference range (<2.5 pmol/L) and two participants were excluded for having serum levels of fT4 below the reference range (10.0 pmol/L). Of the remaining 268 participants, those with thyroid status parameters (where appropriate log transformed) that deviated more than three standard deviations from the population mean were excluded. In total, 259 participants were included, of which 254 had passed away on the 1^st^ of February 2014. The median follow up time was 3.84 years, with interquartile range of 1.90 to 5.89 years.

### Chemical analyses

For both the Leiden Longevity Study and the Leiden 85-plus Study, non-fasted blood samples were collected from all participants between 09:30 hours and 17:00 hours at the assessment visit. Measurements of TSH, fT4 and fT3 were performed using the Modular E170 from Roche, Almere, the Netherlands. The reference ranges at our laboratory are 0.3-4.8 mU/L for TSH (detection range 0.005-100 mU/L), 10.0-24.0 pmol/L for fT4 (detection range 1.3-100 pmol/L) and 2.5-5.5 pmol/L for fT3 (detection range 0.400-50.0 pmol/L). High-sensitivity C-reactive protein (hsCRP) (detection range 0.3-350 mg/L) was measured using Cobas Integra 800 from Roche, Almere, the Netherlands. All coef-ficients of variation were below 5%. The measurements were performed in a single batch at the Department of Clinical Chemistry of the Leiden University Medical Centre.

### Composite measurements of thyroid status

To assess the thyroid status in more detail, three composite measurements were computed. The product of fT4 and TSH (fT4xTSH) was calculated, to allow for assessment of the sensitivity of the pituitary thyrotrophs to feedback regulation by thyroid hormones as des-cribed previously [11, 28]. Furthermore, the ratio of fT4 and TSH (fT4/TSH) was calculated, as a measure of responsiveness of the thyroid gland to TSH as proposed by Jansen et al. [11]. The ratio of fT3 and fT4 (fT3/fT4) was calculated as a measure of thyroid hormone conversion.

### Statistical analyses

Because of the study design of the Leiden Longevity Study in which the men are younger than the women, all analyses were performed on men and women separately. Means and standard deviations, and where appropriate medians and interquartile ranges, were computed for parameters of thyroid status and age and hsCRP for male and female participants of both the Leiden Longevity Study and the Leiden 85-plus Study. Differences between men and women were tested using the T-test, Chi-Square test, or Mann-Whitney U test. Secondly, the participants of both studies were divided over sex-specific tertiles based on TSH, fT4, fT3, the fT4xTSH product, the fT4/TSH ratio, or the fT3/fT4 ratio. Survival analyses were performed using Cox regression on combined tertiles of men and women in both studies and in men and women separately using two statistical models. In model 1 for the Leiden Longevity Study, the analyses were adjusted for differences in age at entry by left truncated analysis and adding age at entry as an independent variable. Furthermore, robust standard errors were used to correct for siblingship by clustering on family number of the Leiden Longevity Study participants. For the Leiden 85-plus Study, model 1 was a crude analysis. In model 2, hsCRP was added as an independent variable, to adjust for inflammation, for both the Leiden Longevity Study and the Leiden 85-plus Study. The outcomes were presented as hazard ratios (HRs) for tertiles with the lowest tertile as a reference. To formally test whether the associations between parameters of thyroid status and mortality were similar for nonagenarians of long-lived families and the general population, interaction between study population (long-lived families or the general population) and parameters of thyroid status on mortality was tested by adding an interaction term and population of origin as covariates to the model 2 analysis excluding familial clustering. To assess a generalizable result for nonagenarian populations, the results were analyzed using a fixed-effects inverse-variance weighted analysis based on the coefficient for the highest tertile compared to the lowest tertile and its Standard Error in Cox regression. The output was transformed to HR. As sensitivity analyses we perform-ed the same analyses in participants with hsCRP <10 mg/L, in participants who had passed away in the first two years of follow up, in participants who had survived the first year of follow up, and in participants with TSH levels within the normal range. In the analyses values of P<0.05 were considered significant. For data analyses Statistical Package for the Social Sciences (SPSS) version 23, the Stata Data analysis and Statistical Software for Windows version 12.0 SE and R version 3.2.4 for Windows were used. The graph was created using GraphPad Prism version 6.05 for Windows.

## SUPPLEMENTARY MATERIAL TABLES



## References

[1] Chaker L, Bianco AC, Jonklaas J, Peeters RP (2017). Hypothyroidism. Lancet.

[2] Franklyn JA, Boelaert K (2012). Thyrotoxicosis. Lancet.

[3] Vestergaard P, Mosekilde L (2003). Hyperthyroidism, bone mineral, and fracture risk—a meta-analysis. Thyroid.

[4] Lee JJ, Pedley A, Marqusee E, Sutherland P, Hoffmann U, Massaro JM, Fox CS (2016). Thyroid function and cardiovascular disease risk factors in euthyroid adults: a cross-sectional and longitudinal study. Clin Endocrinol (Oxf).

[5] Bowers J, Terrien J, Clerget-Froidevaux MS, Gothié JD, Rozing MP, Westendorp RG, van Heemst D, Demeneix BA (2013). Thyroid hormone signaling and homeostasis during aging. Endocr Rev.

[6] von Faber M, Bootsma-van der Wiel A, van Exel E, Gussekloo J, Lagaay AM, van Dongen E, Knook DL, van der Geest S, Westendorp RG (2001). Successful aging in the oldest old: who can be characterized as successfully aged?. Arch Intern Med.

[7] Gussekloo J, van Exel E, de Craen AJ, Meinders AE, Frölich M, Westendorp RG (2004). Thyroid status, disability and cognitive function, and survival in old age. JAMA.

[8] Schoenmaker M, de Craen AJ, de Meijer PH, Beekman M, Blauw GJ, Slagboom PE, Westendorp RG (2006). Evidence of genetic enrichment for exceptional survival using a family approach: the Leiden Longevity Study. Eur J Hum Genet.

[9] Westendorp RG, van Heemst D, Rozing MP, Frölich M, Mooijaart SP, Blauw GJ, Beekman M, Heijmans BT, de Craen AJ, Slagboom PE, Leiden Longevity Study Group (2009). Nonagenarian siblings and their offspring display lower risk of mortality and morbidity than sporadic nonagenarians: The Leiden Longevity Study. J Am Geriatr Soc.

[10] Rozing MP, Houwing-Duistermaat JJ, Slagboom PE, Beekman M, Frölich M, de Craen AJ, Westendorp RG, van Heemst D (2010). Familial longevity is associated with decreased thyroid function. J Clin Endocrinol Metab.

[11] Jansen SW, Akintola AA, Roelfsema F, van der Spoel E, Cobbaert CM, Ballieux BE, Egri P, Kvarta-Papp Z, Gereben B, Fekete C, Slagboom PE, van der Grond J, Demeneix BA (2015). Human longevity is characterised by high thyroid stimulating hormone secretion without altered energy metabolism. Sci Rep.

[12] Nicoloff JT, Lum SM, Spencer CA, Morris R (1984). Peripheral autoregulation of thyroxine to triiodothyronine conversion in man. Horm Metab Res Suppl.

[13] Visser TJ, Peeters RP, De Groot LJ, Beck-Peccoz P, Chrousos G, Dungan K, Grossman A, Hershman JM, Koch C, McLachlan R, New M, Rebar R, Singer F, Vinik A, Weickert MO (2000). Metabolism of Thyroid Hormone. Endotext.

[14] de Vries EM, Fliers E, Boelen A (2015). The molecular basis of the non-thyroidal illness syndrome. J Endocrinol.

[15] Waring AC, Arnold AM, Newman AB, Bùzková P, Hirsch C, Cappola AR (2012). Longitudinal changes in thyroid function in the oldest old and survival: the cardiovascular health study all-stars study. J Clin Endocrinol Metab.

[16] Yeap BB, Alfonso H, Hankey GJ, Flicker L, Golledge J, Norman PE, Chubb SA (2013). Higher free thyroxine levels are associated with all-cause mortality in euthyroid older men: the Health In Men Study. Eur J Endocrinol.

[17] Cappola AR, Arnold AM, Wulczyn K, Carlson M, Robbins J, Psaty BM (2015). Thyroid function in the euthyroid range and adverse outcomes in older adults. J Clin Endocrinol Metab.

[18] van den Beld AW, Visser TJ, Feelders RA, Grobbee DE, Lamberts SW (2005). Thyroid hormone concentrations, disease, physical function, and mortality in elderly men. J Clin Endocrinol Metab.

[19] Taylor PN, Richmond R, Davies N, Sayers A, Stevenson K, Woltersdorf W, Taylor A, Groom A, Northstone K, Ring S, Okosieme O, Rees A, Nitsch D (2016). Paradoxical Relationship Between Body Mass Index and Thyroid Hormone Levels: A Study Using Mendelian Randomization. J Clin Endocrinol Metab.

[20] Flicker L, McCaul KA, Hankey GJ, Jamrozik K, Brown WJ, Byles JE, Almeida OP (2010). Body mass index and survival in men and women aged 70 to 75. J Am Geriatr Soc.

[21] Pearce SH, Razvi S, Yadegarfar ME, Martin-Ruiz C, Kingston A, Collerton J, Visser TJ, Kirkwood TB, Jagger C (2016). Serum Thyroid Function, Mortality and Disability in Advanced Old Age: the Newcastle 85+ Study. J Clin Endocrinol Metab.

[22] Ceresini G, Marina M, Lauretani F, Maggio M, Bandinelli S, Ceda GP, Ferrucci L (2016). Relationship Between Circulating Thyroid-Stimulating Hormone, Free Thyroxine, and Free Triiodothyronine Concentrations and 9-Year Mortality in Euthyroid Elderly Adults. J Am Geriatr Soc.

[23] Ceresini G, Ceda GP, Lauretani F, Maggio M, Usberti E, Marina M, Bandinelli S, Guralnik JM, Valenti G, Ferrucci L (2013). Thyroid status and 6-year mortality in elderly people living in a mildly iodine-deficient area: the aging in the Chianti Area Study. J Am Geriatr Soc.

[24] Pereg D, Tirosh A, Elis A, Neuman Y, Mosseri M, Segev D, Lishner M, Hermoni D (2012). Mortality and coronary heart disease in euthyroid patients. Am J Med.

[25] Rodondi N, Newman AB, Vittinghoff E, de Rekeneire N, Satterfield S, Harris TB, Bauer DC (2005). Subclinical hypothyroidism and the risk of heart failure, other cardiovascular events, and death. Arch Intern Med.

[26] Formiga F, Ferrer A, Padros G, Contra A, Corbella X, Pujol R, Octabaix Study Group (2013). Thyroid status and functional and cognitive status at baseline and survival after 3 years of follow-up: the OCTABAIX study. Eur J Endocrinol.

[27] Schalin-Jäntti C, Ojala AK, Pitkälä KH, Tilvis RS, Strandberg TE (2013). Thyroid-stimulating hormone and mortality in older people. J Am Geriatr Soc.

[28] Yagi H, Pohlenz J, Hayashi Y, Sakurai A, Refetoff S (1997). Resistance to thyroid hormone caused by two mutant thyroid hormone receptors beta, R243Q and R243W, with marked impairment of function that cannot be explained by altered in vitro 3,5,3′-triiodothyroinine binding affinity. J Clin Endocrinol Metab.

[29] Kaneshige M, Suzuki H, Kaneshige K, Cheng J, Wimbrow H, Barlow C, Willingham MC, Cheng S (2001). A targeted dominant negative mutation of the thyroid hormone alpha 1 receptor causes increased mortality, infertility, and dwarfism in mice. Proc Natl Acad Sci USA.

[30] Atzmon G, Barzilai N, Surks MI, Gabriely I (2009). Genetic predisposition to elevated serum thyrotropin is associated with exceptional longevity. J Clin Endocrinol Metab.

[31] He YH, Chen XQ, Yan DJ, Xiao FH, Liu YW, Lin R, Liao XP, Cai WW, Kong QP (2015). Thyroid Function Decreases with Age and May Contribute to Longevity in Chinese Centenarians' Families. J Am Geriatr Soc.

[32] Jansen SW, Roelfsema F, van der Spoel E, Akintola AA, Postmus I, Ballieux BE, Slagboom PE, Cobbaert CM, van der Grond J, Westendorp RG, Pijl H, van Heemst D (2015). Familial Longevity Is Associated With Higher TSH Secretion and Strong TSH-fT3 Relationship. J Clin Endocrinol Metab.

[33] Haentjens P, Van Meerhaeghe A, Poppe K, Velkeniers B (2008). Subclinical thyroid dysfunction and mortality: an estimate of relative and absolute excess all-cause mortality based on time-to-event data from cohort studies. Eur J Endocrinol.

[34] Rodondi N, den Elzen WP, Bauer DC, Cappola AR, Razvi S, Walsh JP, Asvold BO, Iervasi G, Imaizumi M, Collet TH, Bremner A, Maisonneuve P, Sgarbi JA, Thyroid Studies Collaboration (2010). Subclinical hypothyroidism and the risk of coronary heart disease and mortality. JAMA.

