# Supplementary material for: Thyroid status and mortality in nonagenarians from long-lived families and the general population
Source: Aging (Albany NY). 2017 Oct 25;9(10):2223–34. doi: 10.18632/aging.101310 (PMC5680564; doi:10.18632/aging.101310)
Supplement: Supplementary file 1 [file aging-09-2223-s001.pdf]

SUPPLEMENTARY MATERIAL

Table S1. Interaction between parameters of thyroid status and population of origin on mortality.

Parameter of thyroid status	Interaction P-value
TSH	0.70
fT4	0.97
fT3	0.74
fT4xTSH	0.82
fT4/TSH	0.88
fT3/fT4	0.77

Data are presented as P-value for interaction term for parameter of thyroid status and study population on the Cox regression adjusted for age and hsCRP.

Table S2. Parameters of thyroid status and mortality rate in nonagenarians.

Parameter of thyroid status	Hazard ratio (95% CI)	P-value
TSH	0.91 (0.78-1.07)	0.25
fT4	1.22 (1.04-1.43)	0.02
fT3	0.73 (0.62-0.86)	$1.31x10^{-4}$
fT4xTSH	1.00 (0.85-1.17)	0.99
fT4/TSH	1.09 (0.94-1.27)	0.26
fT3/fT4	0.65 (0.55-0.77)	$5.64x10^{-7}$

Data are presented as Hazard ratios for weighted averages of mortality rates of the highest compared to lowest sex-specific tertiles in the Leiden Longevity Study and the Leiden 85-plus Study.
